# Modeling and optimization of simultaneous degradation of rhodamine B and acid red 14 binary solution by homogeneous Fenton reaction: a chemometrics approach

**DOI:** 10.3906/kim-2002-59

**Published:** 2020-08-18

**Authors:** Nasrin ALIASGHARLOU, Morteza BAHRAM, Pezhman ZOLFAGHARI, Naimeh MOHSENI

**Affiliations:** 1 Department of Chemistry, Faculty of Science, Urmia University, Urmia Iran; 2 Department of Chemical Engineering, Faculty of Chemical and Petroleum Engineering, University of Tabriz, Tabriz Iran; 3 Tofigh Daru Research and Engineering Company, Tehran Iran

**Keywords:** Acid red 14, desirability function, fenton reaction, response surface methodology, rhodamine B

## Abstract

This study aimed to propose a mathematical method to investigate and optimize the simultaneous elimination process of multiple organic pollutants using the Fenton process. Hence, the treatment of rhodamine B (RB) and acid red 14 (AR14) dyes in their binary solution was studied. Multivariate curve resolution alternating least square (MCR-ALS), a novel chemometric method, was applied along with correlation constraints to resolute the UV-Vis spectrophotometric data, enabling quantification of investigated dyes despite a high spectral overlapping. Response surface methodology was adopted to assess the model and optimize individual and interactive effects of three independent factors (Fe^2+^, H_2_O_2_ and initial pH) on the simultaneous elimination of RB and AR14. The values of the regression coefficient for RB and AR14 were determined as 98.48 and 98.67 percent, respectively, revealing the reliability of the obtained polynomial models to predict decolorization efficiencies. Desirability function was employed to optimize the independent variables to attain the highest possible degradation performance for both dyes in their binary solution. At the optimum point of operation ([Fe^2+^] = 143.88 mg/L, [H_2_O_2_] = 126.89 mg/L and pH = 3.71), degradation efficiencies of RB and AR14 were found as 81.58% and 80.22%, respectively, which were nearly identical to the experimental results.

## 1. Introduction

Dyes are classified as one of the most significant environmental pollutants whose existence harms the environment and living organisms such as toxicity and carcinogenicity [1]. Annually, 35,000–70,000 metric tons of produced dyes are discharged into natural streams without adequate treatment, which brings up the foremost concern toward dye effluents [2,3].

In recent years, a wide range of wastewater treatment methodologies has been investigated [4–6] to eliminate organic dyes out of which advanced oxidation processes (AOPs) cast a lot of attention due to their prominent advantages. AOPs, based on the production of highly active oxygen species (namely OH^•^, O^−•^_2_ and H_2_O^•^^2^) throughout a chain of chemical reactions, are used to total mineralization of a wide range of persistent organic pollutants. Fenton’s oxidation reaction is one of the most cost-effective AOP methods which uses ferric ions to catalyze the generation of hydroxyl radical for subsequent mineralization organic materials to CO_2_, H_2_O and light-weight organic acids (Eq. (1) and (2)) [7–9].

(1)Fe2++H2O2→Fe3++OH•+OH-

(2)OH•+OrganicCompounds→Degradation Products

Fenton process was affected by various parameters such as the concentration of respective catalysts, concentration of hydrogen peroxide, initial pH value of the solution, temperature, mixing rate and other operational parameters that require the implementation of an optimization strategy to obtain desirable results [10,11]. A handful of methodologies have been explored by researchers to optimize the Fenton process, including but not limited to Taguchi, response surface methodology (RSM), artificial neural network [12,13].

Unfortunately, most of the published works regarding this issue were focused on the optimization of Fenton process for the elimination of a single pollutant rather than an investigation of a more realistic case of study: a mixture of organic pollutants [14]. In such cases, accurate quantification of organic pollutants is of paramount importance. Accordingly, various approaches were implemented by researchers to overcome thisissue. In some studies, spectral overlapping of organic pollutants was ignored and concentration levels were determined at their respective maximum absorption wavelength [15]. Using this method, spectral overlapping of the UV-Vis spectrum of pollutants may result in inaccurate results [16,17]. Another approach was to obtain a single maximum absorption wavelength for the solution, which was widely implemented. Nonetheless, this method reports a single response as degradation efficiency, yet the precision of results is in doubt [18,19]. Some researchers calculated the degradation efficiency of organic pollutants by the means of chemical oxygen demand (COD) and total organic carbon (TOC) [20–23]. However, these approaches were unable to quantify organic pollutants independently in multicomponent systems. Additionally, a contribution from smaller organic intermediates may lead to inaccurate results [24,25].

Chemometric approaches, such as partial least squares (PLS) and multivariate curve resolution alternating least squares (MCR-ALS), are among alternative methods of investigation of spectrophotometric data obtained from multicomponent systems with considerable spectral overlapping [16,26,27]. While some researchers applied the PLS method to quantify organic pollutants, this method suffers various drawbacks, including a high number of calibration data set and its limited effectiveness in the identification and calculation of byproducts during degradation processes [28]. On the contrary, MCR-ALS is a far efficient choice, due to its unique ability of both qualifying and quantifying an analyte in the presence of unknown components [16,29].

The present work aims to study the simultaneous degradation of a binary solution of cationic and anionic dyes of rhodamine B (RB) and acid red 14 (AR14) by homogeneous Fenton process. The physiochemical specification of RB and AR14 dyes are tabulated in Table 1. MCR-ALS technique was applied to the deconvoluted UV-Vis spectra of the solution during the degradation process and attain respective concentration profiles. Response surface methodology, based on central composite design (CCD), was utilized for modeling of the process concerning three independent variables, including concentrations of ferrous ion, hydrogen peroxide and initial pH value. Finally, taking advantage of Derringer’s desirability function, which was widely utilized for the optimization of multiresponse industrial processes, a unique optimum point was acquired.

**Table 1:** Caracteristics of rhodamine B and acid red 14.

Dye name	Chemical structure	Molecular formula	CI number	λ_max_	MW
Rohdamine B	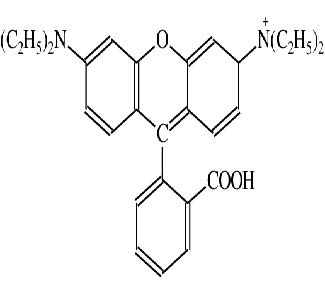	C_28_H_31_C_l_N_2_O_3_	45170	544	479.02
Acid red 14	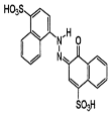	C_20_H_12_N_2_Na_2_O_7_S_2_	14720	515	502.44

## 2. Materials and methods

### 2.1. Reagents and solutions

H_2_O_2_, FeSO_4_.7H_2_O, H _ 2_ SO_4_ , AR14 and RB were purchased from Merck KGaA (Darmstadt, Germany) and used without any further purification. All chemicals were of analytical grade and solutions were prepared in deionized water. Moreover, a 0.1 M solution of H_2_SO_4_ was used to adjust the initial pH of the dye solutions. Stock solutions of FeSO_4_ (4.4 g/L) and H_2_O_2_ (3% w/v) were prepared by dissolving appropriate amounts of them in deionized water.

### 2.2. Instrumentation and software

The pH values of solutions were determined using a Metrohm 780 pH-meter (Metrohm AG, Herisau, Switzerland). An Agilent (Agilent Technologies, Inc., Santa Clara, CA, USA) 8453 spectrophotometer with diode array detection was utilized to record the spectra of solutions from 350 to 650 nm at 1 nm intervals. A graphical user interface for MCR-ALS calculations was used which is available for free. MCR-ALS calculations were performed in MATLAB 7.5 (MathWorks, Natick, MA, USA). Minitab 16.2.4 (Minitab Inc., State College, PS, USA) software was used to design experiments, obtain the model and plot response surfaces.

### 2.3. Working procedure

A 10 mL glass container was used to prepare RB (3.6 mg/L) and AR14 (25 mg/L) binary solutions with a desired amount of FeSO_4_ and H_2_O_2_ solutions following CCD. Afterward, a proper amount of this solution was transferred to a conventional 1 cm path-length cuvette, where the reaction was monitored spectrophotometrically at the intervals of 30 s. In this study, experiments were carried out at room temperature of 25 °C.

### 2.4. Experimental design

After a number of preliminary experiments, a three-level CCD based on three independent parameters (initial dosage (μL) of Fe^2+^ solution (x_1_), initial dosage (μL) of H2 O2 solution (x_2_) and initial pH value of the solution (x_3_)) was designed to model the process using RSM methodology. In this research, initial values of pH, ferrous and hydrogen peroxide dosages were studied in the ranges of 2.5–5.5, 50–250 μl ([Fe^2+^ ] = 55–275 mg/L) and 50–250 μl ([H_2_O_2_] = 37.5–187.5 mg/L), respectively. Afterwards, each parameter was converted to the dimensionless variable (Xi) using Eq. (3) and coded at five levels corresponding to –1.68, –1, 0, 1, and 1.68. Table 2 represents the independent parameters and their respective values at each level.

**Table 2:** Range and level of variables in central composite design along with their real values.

	
		–1.682	-1	0	1	1.682
X_1_: Fe^2+^ (μL)	50	90.54	150	209.45	250
X_2_: H_2_O_2_ (μL)	50	90.54	150	209.45	250
X_3_: pH	2.5	3.1	4	4.89	5.5

(3)Xi=xi-x0δx

A total number of 20 experiments (N) were performed to model the process using RSM. This specific number of experiments was calculated using Eq. (4), which included 8 factorial points, 6 star points and a center point with five additional replications.

(4)N=2k+2k+n0

Where k represents the number of factors and n_0_ is the number of replications.

The results obtained from decolorization experiments can be used to develop a full quadratic equation [Eq. (5)] based on the experimental decolorization data and corresponding parameters and obtain a predictive model for degradation of each dye:

(5)Y=b0+b1X1+b2X2+b3X3+b11X12+b22X22+b33X32+b12X1X2+b13X1X3+b23X2X3

The predicted response *Y* is correlated to the main independent parameters (X_1_, X_2_ and X_3_) by a set of coefficients including intercept (b_0_), linear terms (b_1_, b_2_ and b_3_) , quadratic terms (b_11_, b_22_ and b_33_) and interaction terms (b_12_, b_13_ and b_23_) .

### 2.5. Desirability function

In a multiresponse process, the determination of a unique and global optimum point of operation with respect to all desired responses is a challenging step. Desirability optimization methodology, introduced by Derringer and Suich, is a promising choice for the simultaneous optimization of various functions of a process. In this methodology, responses are converted into dimensionless desirability values called di , in which desirability values close to zero indicates an undesirable quality, whereas values close to unity signal more desirable regions [30,31]. The global desirability of the process can be obtained using Eq. (6), paving the way for calculation of a single optimum point.

(6)Df=d1*d2*...*dnn

## 3. Results and discussions

### 3.1. MCR-ALS Analysis

**Figure 2:**
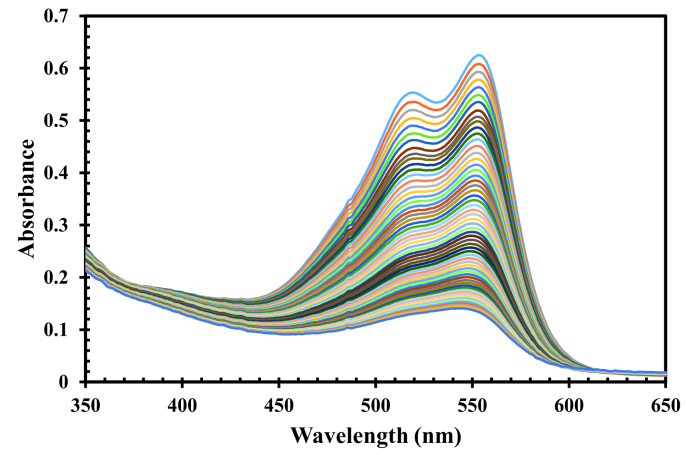
Kinetic spectra recorded during the degradation process of binary mixture of RB and AR14; [RB]_0_ = 3.6 mg/L and [AR14]_0_ = 25 mg/L.

The UV-Vis spectra of RB and AR14 was featured high spectral overlapping at the previously specified concentration demonstrated in Figure 1, which cannot be identified by univariate spectrophotometric approaches. The second-order kinetic-spectrophotometric data obtained from the degradation process of the dyes in their mixture was depicted in Figure 2. As it was shown, after the degradation process there are also some remained species absorbing UV light which cannot be determined due to the shape of spectra.

**Figure:**
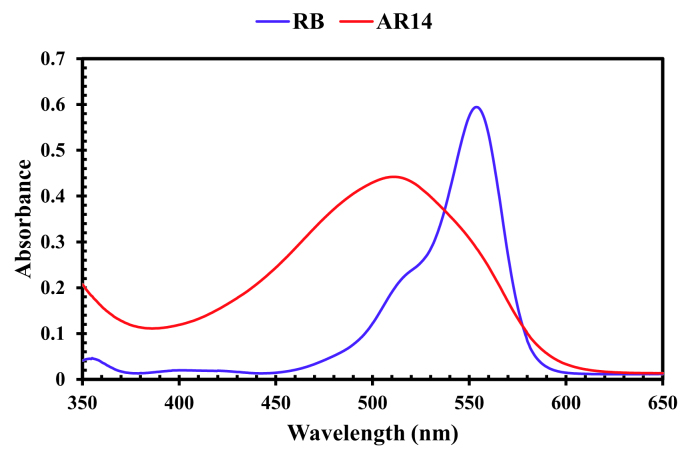
Pure UV-Vis spectra of RB and AR14; [RB] = 3.6 mg/L, [AR14] = 25 mg/L.

MCR-ALS, a multivariate curve resolution technique, uses an iterative algorithm to decompose a twoway data matrix (M) to real and chemically meaningful bilinear models consisting of pure contributions of each component in spectral and concentration directions with a minimal preliminary data regarding the investigated system, even in the presence of unknown interferences [Eq. (7)].

(7)M=CST+E

Where C is the matrix of species’ concentrations, S^T^ is their corresponding pure spectra at the initial dye concentration, and E is the part of data that cannot be expressed by the model and contains an experimental error and/or noise. The ALS algorithm uses initial estimates of C_0_ or S^T^_0_ and optimizes them iteratively by least-square solution and the implementation of proper constraints such as nonnegativity, unimodality, equality and closure to find the best answers [32,33]. The optimized concentration profiles for each component can be applied to calculate the degradation efficiency values (Q) at each experiment using Eq. (8):

(8)Q=s0-ses0

Where S_0_ and Ssub>e are the values of the optimized concentration profiles at the beginning and at an arbitrarily selected time of reaction, respectively. The obtained Q values were used as input responses in our experimental design.

The correct decomposition of the data matrix using MCR-ALS depends on the proper choice of the number of components responsible for variation in the data matrix. Consequently, it is convenient to use eigenvalues obtained by the application of singular value decomposition (SVD) on the data. SVD is a mathematical approach for unique decomposition of a rectangular data matrix ( *R* ) to the product of three smaller matrices *U* , *S* and *V^T^* as presented by Eq. (9):

(9)R=USVT

Where U and V^T^ are orthogonal matrices containing eigenvectors of R and S, including eigenvalues of R on its diagonal. Plotting of the eigenvalues (or relative successive eigenvalues) against the number of components is a diagnostic technique to determine the number of components. The three significant eigenvalues were observed in all data matrices related to the degradation of the mixture of the dyes.

**Figure 3:**
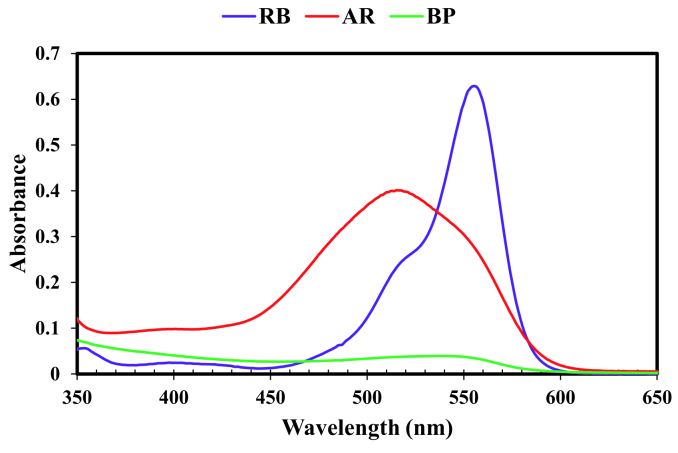
Optimized MCR-ALS spectrum of the investigated dye mixture and the new compound formed during the Fenton process.

There are two main components (RB, AR14) involved in the reaction, while the third component was associated with other molecules that present in the solution such as degradation products including lightweight organic acids [34,35]. In order to initiate the optimization step of MCR-ALS, pure spectra of each component and the degradation spectrum of dyes in their mixture after 30 min of operation, were selected as an initial estimation of dyes and byproduct (BP), respectively. Moreover, nonnegativity and equality constraints were applied to reduce the intrinsic rotational ambiguity of MCR methods. The optimized concentration profileswere used to calculate the responses of each experiment using Eq. (8). Figure 3 exhibits the optimized spectral profile after data treatment by MCR-ALS.

### 3.2. RSM modeling

In order to study the effects of three independent variables on decolorization performance of AR14 and RB dye mixture, a three-factor CCD composed of 20 experiments, was designed and carried out. Experimental results and predicted decolorization performances obtained through RSM modeling are tabulated in Table 3. By the aid of RSM methodology, second-order polynomial equations [Eqs. (10 and 11)] were obtained to predict the decolorization of RB and AR14 and dyes simultaneously in the mixture. In these equations, Y_RB_ and Y_AR14_ represent the decolorization efficiency of RB and AR14 dyes after 30 min of the reaction time, respectively.

(10)YRB=79.52-4.60X1+3.47X2-4.62X3-8.77X12-6.11X22-6.67X32-0.54X1X2+1.65X1X3-0.23X2X3

(11)YAR14=77.80-5.61X1+4.30X2-4.80X3-7.49X12-6.34X22-8.24X32+0.12X1X2--0.07X1X3+0.11X2X3

**Table 3:** The experimental CCD design and actual responses of the dye degradation.

Exp. #	Coded levels	AR14 degradation (%)	RB degradation (%)
				X_1_	X_2_	X_3_	Exp.	Pred.	Exp.	Pred.
1	–1.682	0	0	65.41	66.03	61.12	62.44
2	-1	-1	-1	63.01	61.99	65.37	65.04
3^(CP)^	0	0	0	74.25	77.80	76.87	79.52
4	0	1.682	0	65.17	67.10	69.44	68.08
5 ^(CP)^	0	0	0	79.26	77.80	80.89	79.52
6^(CP)^	0	0	0	78.74	77.80	79.95	79.52
7^(CP)^	0	0	0	77.64	77.80	80.18	79.52
8	0	–1.682	0	52.40	52.62	54.33	56.40
9	1	1	-1	61.42	59.30	57.08	59.00
10^(CP)^	0	0	0	78.27	77.80	79.58	79.52
11	1	-1	–1	51.73	50.66	55.92	53.62
12	0	0	–1.682	59.98	62.56	67.21	68.37
13	1	-1	1	40.81	40.67	46.06	47.21
14	-1	-1	1	51.71	52.30	54.45	52.01
15	-1	1	-1	71.53	70.13	74.28	72.61
16^(CP)^	0	0	0	79.02	77.80	79.81	79.52
17	0	0	1.682	46.82	46.39	53.27	52.82
18	-1	1	1	61.36	60.89	58.73	60.52
19	1.682	0	0	45.61	47.14	47.56	46.95
20	1	1	1	50.28	49.76	53.71	53.53

^(CP)^ indicates central points.

**Figure 4:**
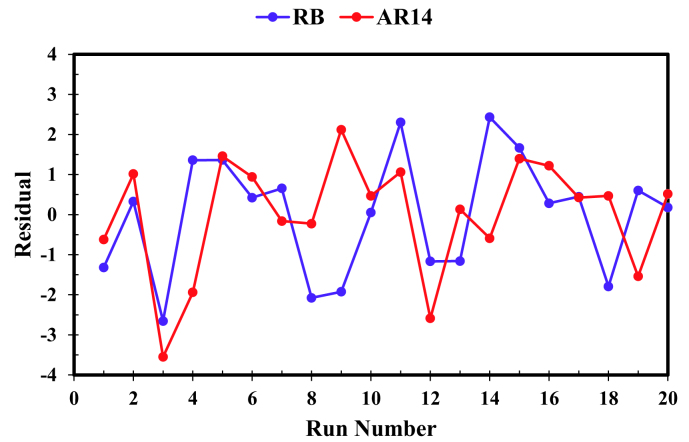
Residual plots obtained from CCD.

**Figure 5:**
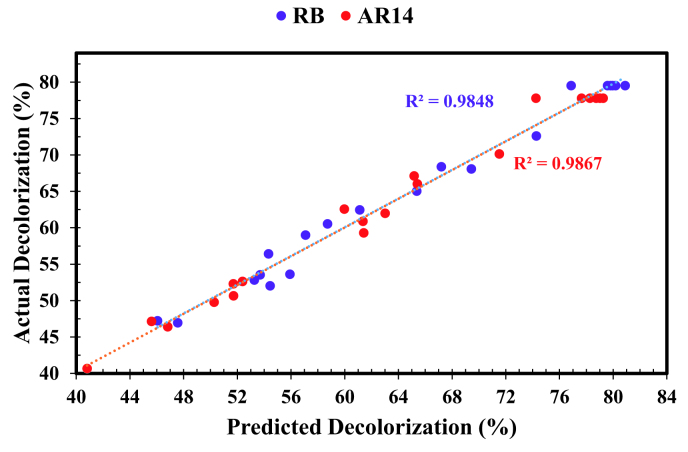
Predicted and experimental degradation efficiency of the dye mixture of AR14 (a) and RB (b).

Residuals, defined as the difference between the predicted and actual values of each run, can be used toinvestigate the reliability of these models. The distribution of residual among the performed experiments is demonstrated in Figure 4. Random distribution of residuals, observed in this figure, signals the reliability of proposed models [36]. Figure 5 presents the normal probability plot of residuals of simultaneous RB and AR14 degradation process. According to this figure, residuals are distributed in a nearly linear form, providing support for the reasonability of the proposed models.

Analysis of variances (ANOVA) technique was employed to further investigate the statistical significance of the presented models. The results of this analysis were tabulated in Tables 4 and 5 for RB and AR14 dyes, respectively. The regression coefficients (R^2^) were found to be 0.9848 and 0.9867, meaning that 98.48 and 98.67 percent of variances were predictable by the presented models for RB and AR14 dyes, respectively. Meanwhile, the R^2^_adj_ values for RB and AR14 decolorization process were found as 0.971 and 0.9747, which were close to their respective regression coefficient. The neglectable difference between R^2^ and R^2^_adj_ values further confirms the adequacy of the obtained mathematical models [37].

**Table 4:** Analysis of variances for RB decolorization in the RB-AR14 mixture by the Fenton process.

Source of variation	DF	SS	Adj. SS	Adj. MS	F value	P value
Regression	9	2700.05	2700.05	300.01	71.80	0.000
Residual error	10	41.78	41.78	4.18		
Lack-of-fit	5	32.18	32.18	6.44	3.35	0.105
Pure error	5	9.60	9.60	1.92		
Total	19	2741.84				
	R^2^ = 98.48%		R^2^(pred) = 89.70%		R^2^(adj) = 97.10%	

**Table 5:** Analysis of variances for AR14 decolorization in the RB-AR14 mixture by the Fenton process.

Source of variation	DF	SS	Adj. SS	Adj. MS	F value	P value
Regression	9	2983.86	2983.86	331.54	82.39	0.000
Residual error	10	40.24	40.24	4.02		
Lack-of-fit	5	22.91	22.91	4.58	1.32	0.383
Pure error	5	17.33	17.33	3.46		
Total	19	3024.10				

Fischer’s test values (F-values) were also reported in Tables 4 and 5. The respective F-values for the degradation model of RB and AR14 were reported as 71.80 and 82.39, combined with a low probability value of 0.000, which demonstrates that both models were significant. On the other hand, F-values of lack of fit were calculated as 3.35 and 1.32 for RB and AR14, respectively, which in conjunction with relatively high probability values of 0.105 and 0.383, proves the insignificancy of lack of fit [38].

### 3.3. Process optimization and effect of variables

In order to optimize this multiresponse process, the desirability function [Eq. (6)] was applied. Desirability values were calculated by considering the minimum and maximum desirable degradation performances of 75 and 85 percent, respectively. At the optimal point of operation with regard to degradation performance of RB and AR14, decolorization efficiencies of 81.58 and 80.22 percent were achieved for RB and AR14 dyes, resulting in a composite desirability value of 0.72. In order to reach this level of effectiveness, the optimal levels of added volumes of Fe^2+^ and H_2_O_2_ and pH value were determined as 130.80 μl ([Fe^2+^] = 143.88 mg/L), 169.19 μl ([H_2_O_2_] = 126.89 mg/L) and 3.71, respectively. Decolorization of RB and AR14 mixture at the abovementioned conditions was performed three times to validate the accuracy of results predicted by the proposed RSM-desirability function method. The average decolorization values of RB and AR14 at the optimum point of operation were found to be 81.32 (σ = 0.30) and 79.69 (σ = 0.53) percent, respectively, which were in a good agreement with the predictions made by the model.

**Figure 6:**
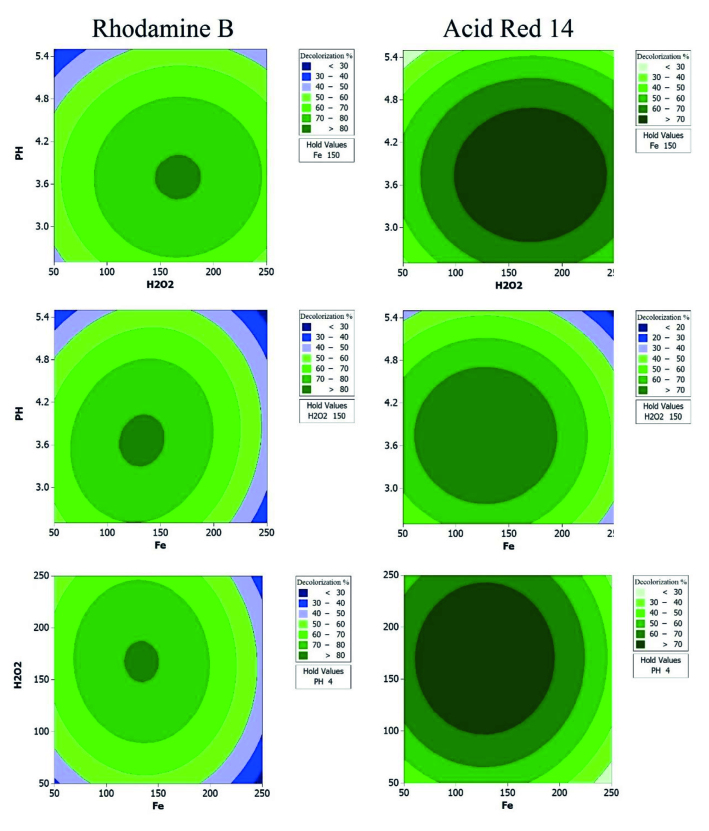
Obtained response contours of simultaneous degradation of RB and AR14 in their binary mixture.

Two-dimensional contour plots based on polynomial models [Eqs. (10) and (11)] were generated and depicted in Figure 6 to thoroughly illustrate the effects of three independent variables on the degradation values of RB and AR14. As it was observed, degradation efficiencies of RB and AR14 were greatly affected by independent variables.

According to Figure 6, the decolorization efficiency of RB and AR14 was highly dependent on Fe^2+^ concentration. Starting from the minimum level of Fe^2+^, decolorization performances of both of the dyes increases until reaching its climax at medium dosages around Fe2+ (around 150 μL), where it begins moving downward. By increasing [Fe^2+^], the production rate of hydroxyl radicals raises as well [Eq. (1)], however, at higher concentrations of Fe^2+^, it may act as a scavenger of hydroxyl radicals and hydroxyl radical production rate declines [Eq. (13)] [37]. Furthermore, excessive production of Fe^3+^ ions as a result of these reactions leads to the generation of per hydroxyl radicals (HO•_2_) by initiating a reaction described by [Eq. (14)] . Compared to hydroxyl, per hydroxyl radicals are less active in the degradation process of organic compounds and by quenching of hydroxyl radicals, the number of effectively available hydroxyl radicals diminishes [Eq. (15)]. Decolorization efficiency of RB and AR14 dyes by Fenton reaction mainly depends on hydroxyl radical’s destructive power and thus, it is necessary to operate at the optimum level of [Fe^2+^] to attain the highest possible performance.

(12)Fe2++H2O2→Fe3++OH•+OH-

(13)Fe2++OH•→Fe3++OH-

(14)Fe3++H2O2→Fe2++HO2•+H+

(15)HO2•+OH•→O2+H2O

From economical and safety points of view, it is essential to optimize hydrogen peroxide dosage in every process involving this reagent, including the Fenton process. As seen in Figure 6, elimination performances of RB and AR14 dyes were suffered drastically from both excessive and deficient dosage of hydrogen peroxide. Generally, Fenton reaction benefits from extra concentrations of hydrogen peroxide, as it accelerates hydroxyl radical generation through [Eq. (1)]. However, a further increment of [H_2_O_2_] beyond a specific critical level may result in lower decolorization efficiencies. At such concentrations, hydrogen peroxide curtails the number of available hydroxyl radicals to attack organic molecules by playing scavenger role. First of all, hydrogen peroxide may directly attack hydroxyl radicals [Eq. (16)], converting them to per hydroxyl radicals [39,40]. Another side effect of the increased level of [H_2_O_2_] is the faster deactivation of ferrous ions to ferric state [Eq. (14)], which accelerates the formation of per hydroxyl radicals. Additionally, per hydroxyl radicals may enter a direct reaction with hydroxyl radicals [Eq. (15)], further reducing the number of available hydroxyl radicals to react with RB, AR14 and other organic molecules, resulting in a lower decolorization efficiency.

(16)H2O2+OH•→HO2•+H2O

It is well known that the efficiency of the Fenton process is directly affected by the pH value of the solution. Many researchers investigated the effects of the pH value of the solution and reported that Fenton reaction performs better at acidic environments, with a pH value around 3–3.5 [41]. In our research, the decolorization performance of RB and AR14 mixture was investigated at pH values ranging from 2.5 to 5.5, with an optimumvalue of 3.71. At higher pH values (basic conditions), hydrogen peroxide’s stability decreases, prohibiting its reaction with ferrous ions [Eq. (1)] to form effective hydroxyl radicals. In addition, the formation of Fe(OH)_3_ in the form of precipitations further decreases the effectiveness of the Fenton reaction [42]. In contrast, hydrogen peroxide forms H_3_O^+^_2_ ions by taking an additional proton in a severe acidic environment, reducing the production rate of hydroxyl radicals. As a result, it is important to detect the optimum pH value to escalate the decolorization rate of organic pollutants by Fenton reaction.

## 4. Conclusion

In this work, simultaneous degradation of a binary mixture of RB and AR 14 dyes in aqueous solution was studied by homogenous Fenton reaction. MCR-ALS method, in combination with UV-vis spectrophotometry, was utilized for quantification of desired analytes in the presence of unknown interferents without performing timeconsuming calibration processes. Next, response surface methodology based on a three-level central composite design was applied to optimize and assess the effects of operational parameters (pH and dosages of Fe^2+^ and H_2_O_2_) on decolorization efficiency during the homogenous Fenton degradation process. Analysis of variances demonstrated high regression coefficients (R^2^ = 98.48 and 98.67 percent for RB and AR14 dyes, respectively), revealing the reliability of the proposed mathematical model and a good agreement between experimental and predicted values. Finally, the desirability function method was applied to determine the best condition to maximize the degradation efficiencies of RB and AR14 simultaneously. The acquired optimum conditions were calculated as [Fe^2+^] = 143.88 mg/l, [H_2_O_2_] = 126.89, and pH value of 3.71. Performing the decolorization experiment under these conditions, degradation efficiencies of RB and AR14 were found to be 81.32 and 79.69 percent, respectively, matching the calculated values by a small margin. Results proved that the combination of the implemented mathematical methodologies presents a fast, reliable and accurate way for monitoring, quantification and simultaneous optimization of organic pollutants in complex processes and mixtures.
